# Status, perspectives and trends of satellite navigation

**DOI:** 10.1186/s43020-020-00023-x

**Published:** 2020-08-03

**Authors:** Guenter W. Hein

**Affiliations:** 1 Emeritus of Excellence, Universität der Bundeswehr München, Werner-Heisenberg-Weg 39, 85577 Neubiberg, Germany; 2 Chairman of the Executive Board, Munich Aerospace e. V., Ludwig Bölkow Campus, Willy-Messerschmitt-Str. 1, 82024 Taufkirchen bei München, Germany

**Keywords:** GPS, GLONASS, BDS, Galileo, Status, Perspectives, Challenges, Megatrends

## Abstract

This paper reviews the status of satellite navigation (as per 11 May 2020)—without claim for completeness—and discusses the various global navigation satellite systems, regional satellite navigation systems and satellite-based augmentation systems. Problems and challenges for delivering nowadays a safe and reliable navigation are discussed. New opportunities, perspectives and megatrends of satellite navigation are outlined. Some remarks are closing this paper emphasizing the great value of satellite navigation at present and in future.

## Introduction

The Global Positioning System (GPS) project was started by the U.S. Department of Defense in 1973, with the first prototype spacecraft of Block 1 launched in 1978. The first of nine satellites in the initial Block II series was launched 1989 and the full constellation of 24 satellites became operational (Full Operational Capability—FOC) in 1993.

Shortly after (1982) the Russian GLONASS system was built up with a first Final Operational Capability (FOC) in 1996. However, due to the short life of the satellites the constellation dropped down in 2002 to as few as seven satellites. In 2011 FOC was achieved again with 24 satellites after having launched approximately 140 satellites in total (Langley 2017).

In the first step, China developed an active positioning system called BeiDou Navigation Demonstration System (BDS-1), which started in 1994 and consisted of two in-orbit Geostationary Earth Orbit (GEO) satellites launched in 2000 and a third one launched in 2003. In the second step, the passive positioning system BeiDou Navigation Satellite (Regional) System (BDS-2) followed between 2004 and 2012 with a total of 14 satellites, including 5 GEO, 5 Inclined Geosynchronous Orbits (IGSO) and 4 Medium Earth Orbit (MEO) satellites serving the Asia–Pacific region. The third step, BeiDou Navigation Satellite System with Global Coverage (BDS-3), is developed between 2004 and 2020. It will be comprehensively completed with the launching of 30 satellites (China Satellite Navigation Office 2019).

The European Union (EU) launched the first In-Orbit Validation (IOV) satellites GIOVE-A and GIOVE-B of the Galileo system 2011. Galileo will be completed end of 2020/beginning of 2021.

It took obviously 20 years to build up the first Global Navigation Satellite System (GNSS), namely GPS, the last one, BDS-3 required only 3 years.

Regional navigation satellite systems (Japan, India) were developed for the sake of joining this new high-tech space world and gaining exclusive access to a system for governmental and military reasons.

Since GPS was first considered as a military system (now it is dual-use), civilian aviation was hesitating to use it for aircraft navigation and landings. Satellite-Based Augmentation Systems (SBAS) were developed all over the world delivering the required integrity of the system via GEO satellites to the user.

We can show now over 47 years of modern satellite navigation: four global systems, two regional systems and a large number of SBAS are available. However, it is only less than one decade that the user started to take advantage. The number of possible GNSS applications is not limited by technology rather than by our imagination. And the development of satellite navigation is not finished. New opportunities are coming up, however, also new threats for a safe and reliable navigation appear.

This paper reviews the state-of-art of the global, regional and augmentation systems. Problems and challenges are discussed and new opportunities, perspectives and megatrends of satellite navigation are outlined.

## Satellite navigation systems

### Global Navigation Satellite Systems (GNSS)

**Fig. 1:**
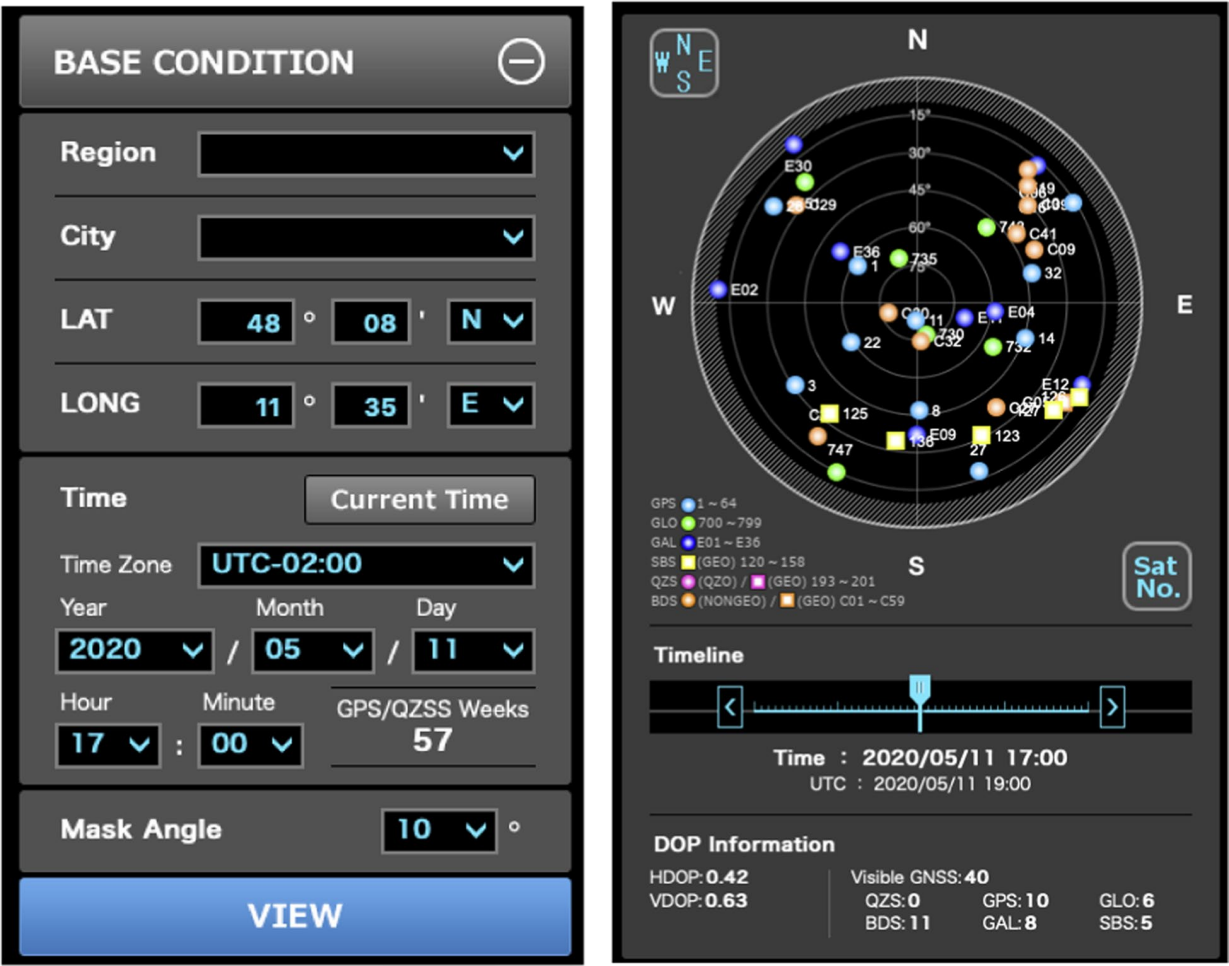
GNSS Satellites in View in Munich on 11 May 2020. Reference: https://qzss.go.jp/en/technical/gnssview/index.html

**Fig. 2:**
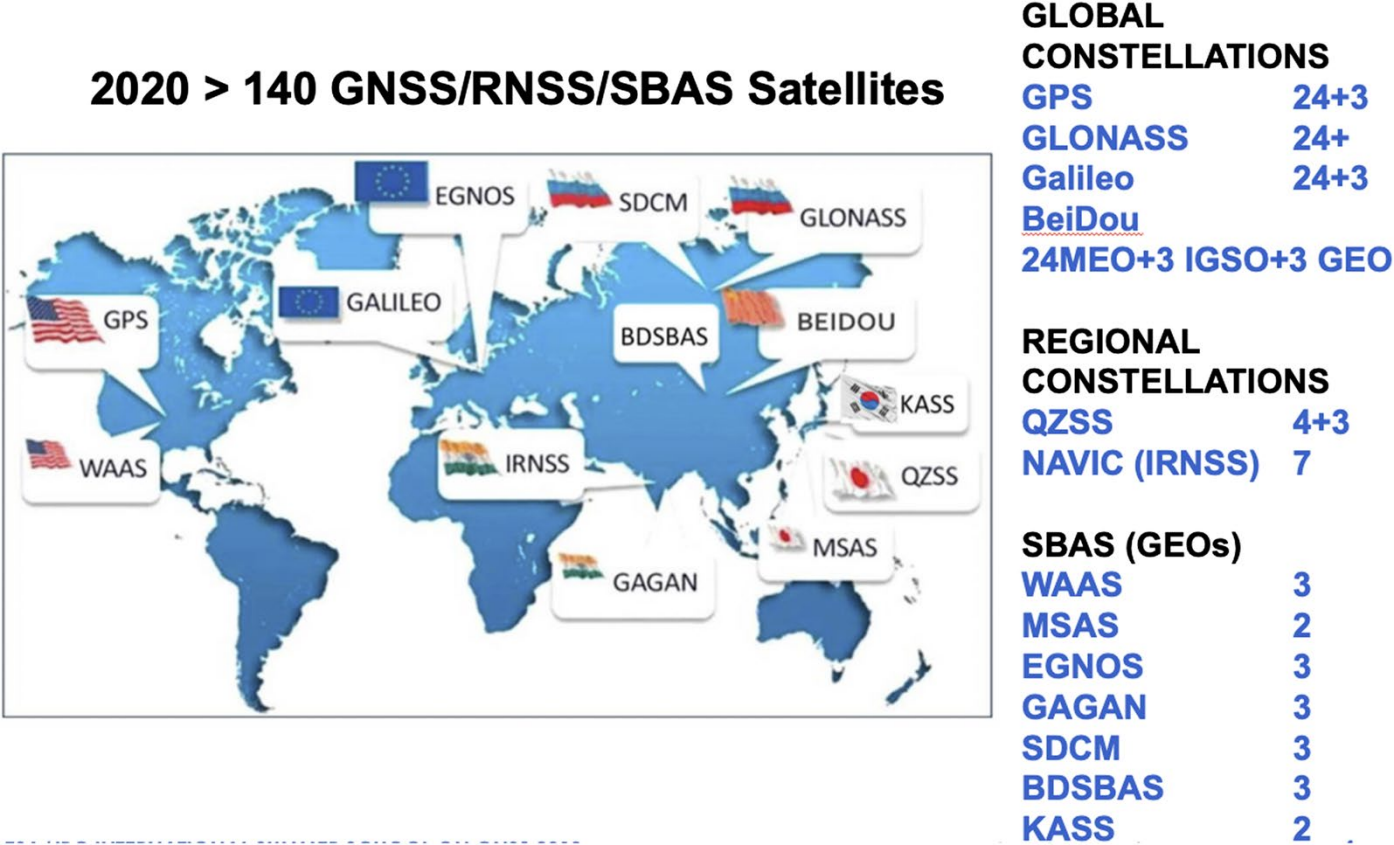
GNSS: a system of systems. (The South Korean System KPS is not considered since developments did not start yet)

Four GNSSs are available for the users, two already fully available, one of them to be finished first half of 2020 (BDS-3) and the other to become fully operational end of 2020/beginning 2021 (Galileo Navigation Satellite System (Galileo)). Assuming an unobstructed view, 35 GNSS satellites could be used on 11 May 2020 in Munich considering a mask angle of 10° (Fig. 1). 5 SBAS satellites are available. A GNSS System of Systems has been built up (Fig. 2).

Three-dimensional positioning and navigation require four satellites in minimum. Thus, even in urban areas we may see some redundancy in satellites. The so-called interoperability (in its most strict sense assuming same center frequencies but different codes) achieved between almost all of the four GNSS may have the drawback that the internal noise floor is increasing causing eventually problems in signal acquisition of the receivers (Hein 2010). According to this, the benefit of various GNSS when the number of its satellites is, let’s say, higher than 24 is questionable since all receivers are anyway of type multi-GNSS ones (at least in the civilian world), no more receivers considering only one system are built and sold, and the civilian user is applying a multi-GNSS positioning. Similar arguments can be found also for the regional satellite navigation systems.

Their main purpose can be only military and/or to follow the high-tech developments in that satellite field. For example, the two regional systems of Japan (QZSS) and of South Korea (KPS) are located so near together that both will be visible for the users in the corresponding countries. As mentioned above, 35–40 satellites (depending on the mask angle) are visible for the user in areas with no obstructions. Do we require so many satellites, do we need four global systems? Four satellites are necessary for three-dimensional positioning and when combining several GNSS, up to three additional satellites for solving the time-offsets in between. What are we doing with that high redundancy in satellite observations? Multiple Receiver Autonomous Integrity Monitoring (RAIM) can be applied in order to control the different satellite observations/systems and advanced multipath mitigation are becoming possible, just to mention two applications. However, there are more possibilities which are not yet really explored!

#### Global Positioning System (GPS)

The first two satellites of the next generation GPS III were launched 23 December 2018 and 22 August 2019 respectively and have successfully completed the in-orbit check. The main new features of the GPS III satellites include increased accuracy and transmission power, inherent signal integrity, the new L1C civil signal and a longer life of 15 years. The launch of the third GPS III satellite is planned for July 2020. Currently (21 April 2020) there are 11 Block IIR, 7 Block IIR-M, 12 Block IIF and 1 Block III satellites operational. The next generation Operational Control System (OCX) is the future version of the GPS control segment which will command all modernized and legacy GPS satellites, manage all civil and military navigation signals, and provide improved cybersecurity and resilience for the next generation of GPS operations. OCX will be ready for transitions to operations mid of 2022 (http://www.gps.gov).

#### GLObal NAvigation Satellite System (GLONASS)

The last GLONASS-M launch took place on 16 March 2020. A new generation of GLONASS-K satellites is under development, with two initial spacecraft already in orbit. Further GLONASS-K launches are expected next year via Soyuz and Proton-M rockets. Main recent changes of the GLONASS system are the introduction of Code Division Multiple Access (CDMA) signals while keeping the Frequency Division Multiple Access (FDMA) signals and the improvement of the on-board clock stability. The future addition of an IGSO regional part (GLONASS-B)—similar to BeiDou—and a better world-wide geographically distributed control network (currently Russia only) are planned. Meanwhile on-board cross-links are used for orbit and clock updates outside the current ground control visibility (http://www.glonass-iac.ru/en/).

#### Galileo Navigation Satellite System (Galileo)

The next two satellites to be launched with Soyuz spacecraft are planned for end of 2020 or beginning of 2021, upgrading the constellation to 24 operational satellites (including three In-Orbit Validation (IOV) satellites), see e.g. http://www.gsc-europa.eu/system-service-status/constellation-information, http://www.gsa.europa.eu/european-gnss/galileo/galileo-european-global-satellite-based-navigation-system, (Chatre and Benedicto 2019). Based on this, the European Union may declare then “full operational capability” depending on how this will be defined. Earlier EC statements were looking for 30 satellites. The Signal-in-Space Error (SISE) of about 0.25 m (95%) achieved in 2019 (Benedicto 2019) is smaller than that of GPS (https://www.nstb.tc.faa.gov/reports/2019_Q4_SPS_PAN.pdf), (Barnes 2019; Lavrakas 2020). However, these values depend on the update rate frequency of Galileo (100 min) versus GPS (12 h).

Galileo experienced an outage from 11 July to 17 July 2019. The 6-day service outage occurred during a system upgrade in the ground infrastructure due to a mishandling of a temporary equipment and follow-on events.

The contract for the first order (Batch 4) of Galileo transition to Galileo Second Generation (G2G) satellites is planned to be placed end of 2020. Batch-3 for in-orbit spares and replacements for the oldest Galileo (IOV) satellites (launched 2011/12) contained 12 satellites. Since then, the decision for a now free-of-charge “commercial service” has been taken and the old commercial service will be replaced by a High-Accuracy Service (HAS) and a Commercial Authentication Service (CAS), expected to become operational in 2020. The HAS will provide Precise Point Positioning (PPP) in E6B and achieve accuracies of 20–40 cm globally, with a 5-min convergence. Additional corrections broadcast regionally in Europe will have target convergence within 100 s.

#### BeiDou Navigation Satellite System (BDS)

Since November 2017, there were 18 successive launches within 2 years. 28 BDS-3 satellites and BDS-2 backup satellites have been successfully injected the last launch on March 9, 2020 carried the 54th BDS satellite and 29th BDS-3 satellite into the designated geosynchronous orbit while the BDS-3 construction has entered the final stage. One more GEO satellite will be launched probably in May 2020 which completes the BDS-3 system about half a year ahead of the scheduled target. The nominal BDS-3 constellation consists of 24 MEO, 3 IGSO and 3 GEO satellites. BeiDou has intersatellite links and provides also a PPP service (Li et al. 2014, 2020; Ruan et al. 2020; Viet et al. 2020; Yang et al. 2020; Zhang et al. 2019; Zhu et al. 2018), (www.en.beidou.gov.cn).

The orbital constellations of the GNSS (as per 11 May 2020) can be found in Table 1.

**Table 1:** GNSS orbital constellations (status 11 May 2020)

GNSS constellation Status 11.05.2020	GPS	GLONASS	Galileo	BDS-2	BDS-3
Total satellites in constellation	32	27	24	15	34
Operational	31	24	22	15	28
Not included in orbital constellation					5
Under maintenance	1				
Spares		2			
In flight test phase		1	2		
IOV SV included in operational constellation			3		

### Regional Navigation Satellite Systems (RNSS)

#### Indian Regional Navigation Satellite System IRNSS/NavIC

**Fig. 3:**
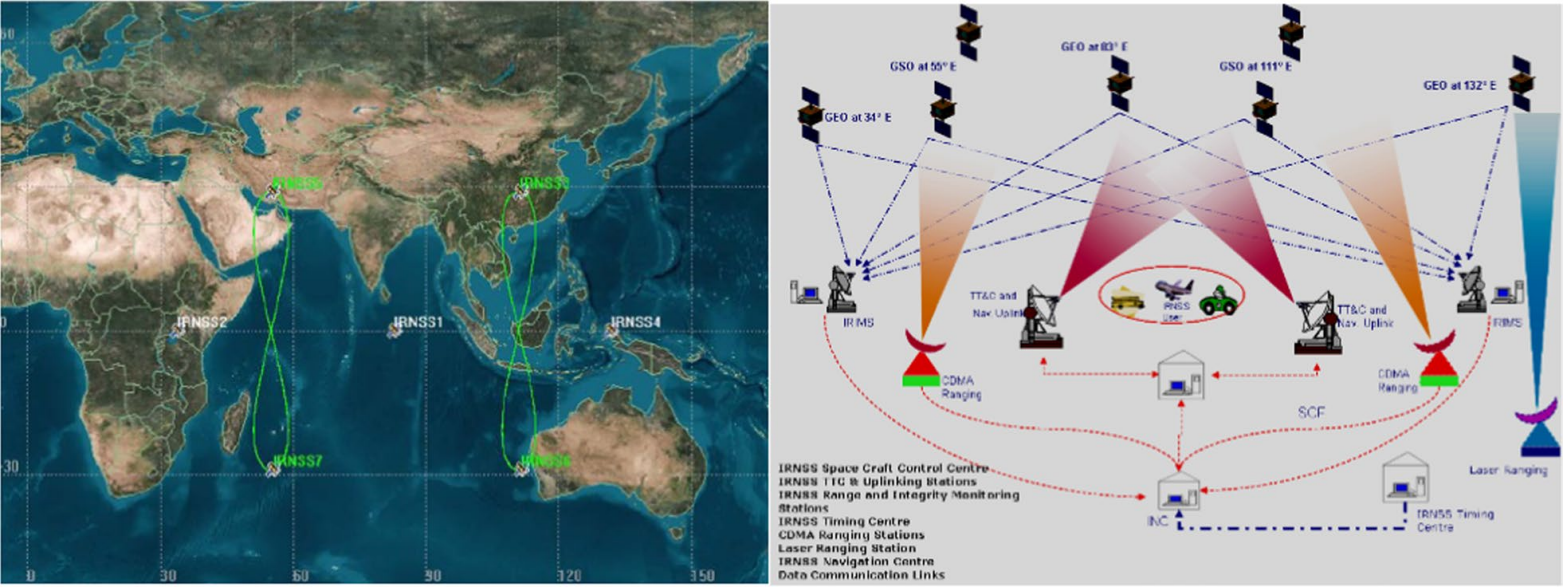
The Indian Regional Navigation Satellite System (IRNSS/NavIC)—Reference: http://www.isro.gov.in

Figure 3 shows the IRNSS/NavIC. The independent Indian satellite-based positioning system for critical national applications has the main objective to provide reliable position, navigation and timing services over India and about 1500 km around India. It has been (re-)named Navigation with Indian Constellation (NavIC) recently. It consists currently of three GEO and five IGSO satellites. In January 2017 a complete failure of IRNSS 1A occurred when all 3 atomic clocks failed. One launch (IRNSS-1H, on 3 August 2017) was unsuccessful the satellite could not reach orbit.

#### Japanese Quasi-Zenith Satellite System (QZSS)

**Fig. 4:**
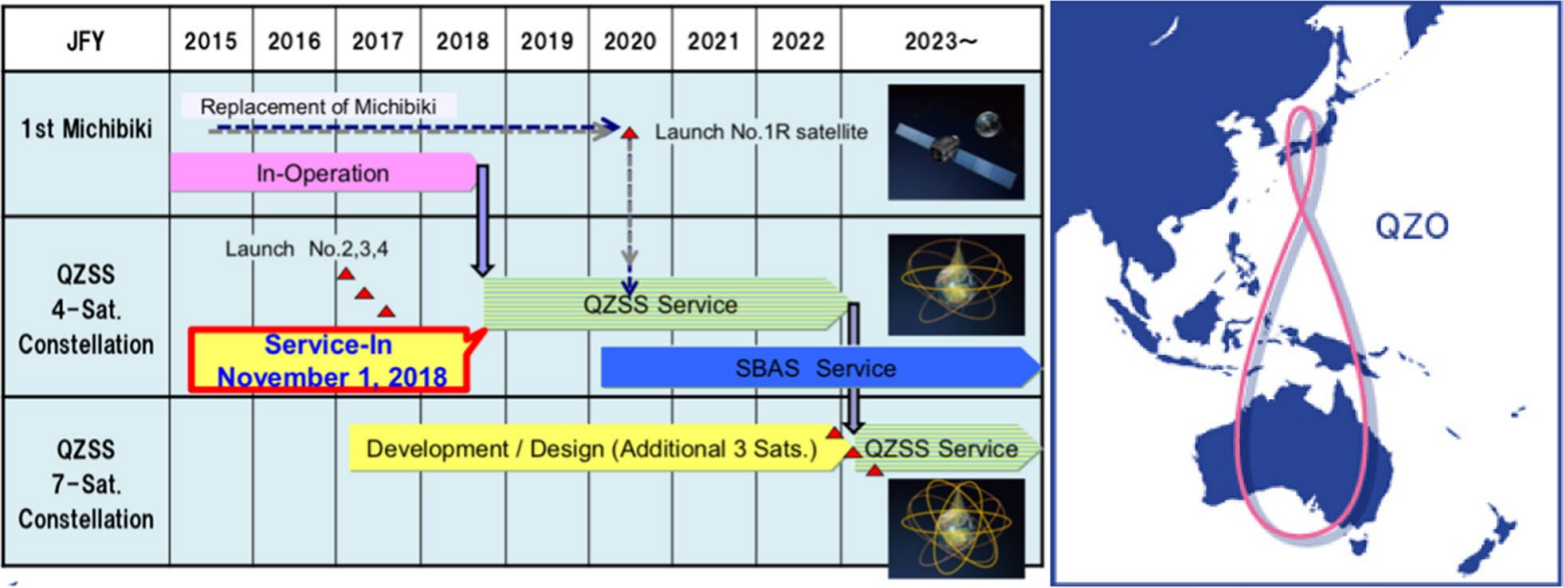
The Japanese Quasi-Zenith Satellite System (QZSS)—Reference: http://qzss.go.jp/en/

Figure 4 shows the QZSS, which is a regional satellite navigation system complement to GPS. It will take over after 2020/23 also the transmission of the Japanese (multifunctional) Satellite-Based Augmentation Systems (SBAS) called Multifunctional Transport Satellites (MTSAT) (or The MTSAT Satellite Augmentation System (MSAS)) serving currently mainly aviation. Three other satellites will be added after 2023 extending the current QZSS with four IGSO satellites.

#### Regional South Korean Positioning System (KPS)

**Fig. 5:**
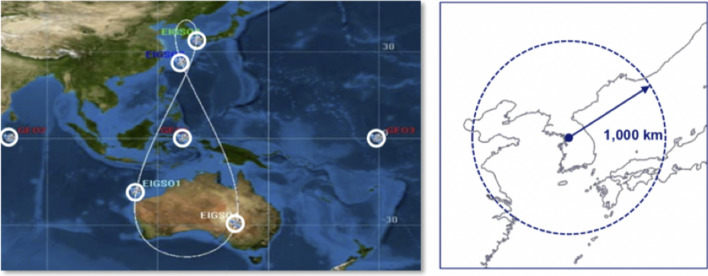
The Regional South Korean Positioning System (KPS)—Constellation and Target Area. Reference: Moonbeom (2019)

In its 3rd Basic Plan for Space Development the South Korean government has decided in February 2018 to plan its own regional satellite navigation system of three GEOs and four elliptical IGSOs (Fig. 5), similar to NavIC and QZSS, covering South Korea and about 1000 km of its surrounding area.

### Satellite-Based Augmentation Systems (SBAS)

**Fig. 6:**
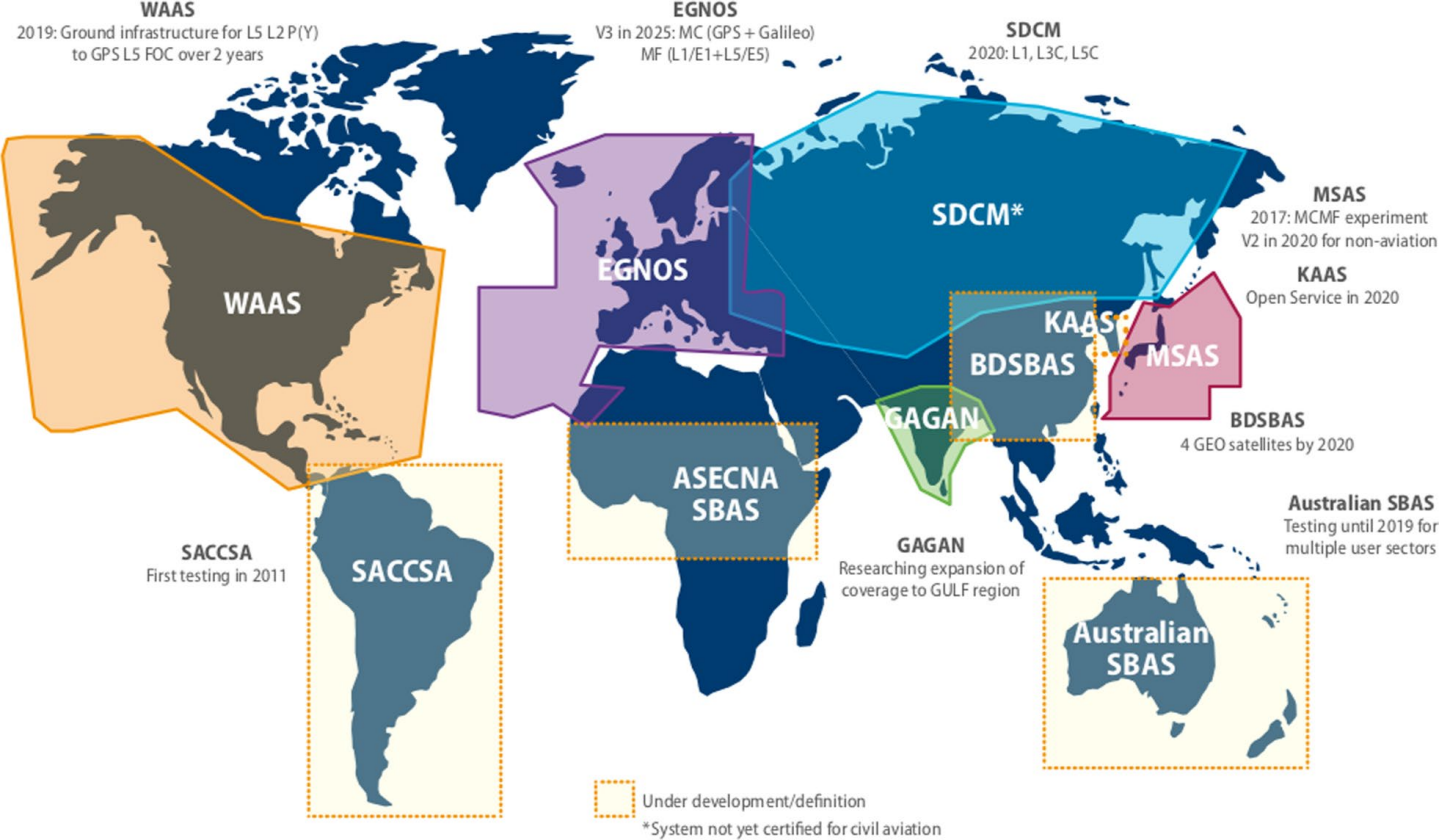
Existing and satellite-based augmentation systems (SBAS) under development. Reference: https://www.gsa.europa.eu/sites/default/files/brochure_o_2017_v6.pdf (figure expanded)

SBAS has the two main purposes: to provide integrity for civil aviation and to transmit differential GNSS and ionospheric corrections. This is achieved by geostationary satellites (in general two to three per SBAS) which are transmitting the so-called integrity message and the corrections. A corresponding ground network covering the SBAS area under consideration determines the integrity of GPS, the differential and ionospheric corrections and uplinks it to the GEOs. Europe is currently developing European Geostationary Navigation Overlay Service (EGNOS) V3, the first world-wide dual-frequency (L1/E1, L5/E5a) dual-system (GPS *and* Galileo) SBAS, to go into operation around 2026 when Full Operational Capability (FOC) of GPS L5 is available. Figure 6 shows global SBAS realised and under development.

## Problems and challenges

*Compatibility.*^1^ There are good reasons that the L-band was mainly taken for satellite navigation systems (all-weather-system). However, the frequencies are all heavily occupied. In the past smart signal processing methods allowed the co-existence of several navigation signals within a certain small neglecting interference level (e.g. < 0.2 dB). There all still intentions to put even more signals on the L-band and GPS/GNSS frequencies. The communication signals of the 5G Ligado broadband network were approved (with the condition “Broadcast till you break it”—the GPS interference Standard) end of April 2020 by the U. S. Federal Communication Commission (FCC) despite the concern of the Department of Defense, the Department of Transportation and many others, see https://www.gpsworld.com/fcc-approves-ligado-broadband-network-dod-and-gps-industry-react/.

For all satellite navigation system providers, it is a serious question how any future evolution on signals may be realized. The possible use of smart signal processing techniques as mentioned above is coming to a limit—no other signals in the L-band are possible. The S-band is already crowded, and using the C-band (investigated already by the U. S. in the 1960s when developing GPS) has more serious drawbacks (more required signal power on the satellite and/or active antennae, influence of rain and snow, larger antennae and higher costs for a receiver) than appreciating the value of a possible increase of accuracy due to smaller wavelengths. Flexibilities in signal generation and transmission at satellite level and user reception may contribute to solve the congestions of the frequency bands by giving (partly) up the backward compatibility in coming evolutions of satellite navigation systems.

*Un*-*intended and intended interferences* (jamming and spoofing) are increasing with every day making it more and more complicated or even impossible to fully protect the safety-of-life and authorized/military signals. GNSS jamming devices can be easily bought by everybody, in particular in the internet. In many countries these may be purchased legally though their use is not permitted. All kinds of intentional and unintentional interferences in the GNSS band can be expected to increase. In addition, spoofing devices are nowadays readily available which in the past were available for military use in NAVigational WARfare (NAVWAR) only. There are some measures undertaken to monitor interferences but these are more on a local and regional scale. GNSS signal authentication is a powerful counterpart to GNSS spoofing. Most of the GNSS receivers are neither equipped with interference and spoofing detection nor mitigation software for those effects. GNSS satellites of the past were not prepared for cyber-attacks.

However, all those developments may have a crucial impact on safety-related applications. Providing secure and trustworthy satellite navigation will be one of the main future challenges (Kaplan 2019; Simsky 2019), (https://www.maritimeglobalsecurity.org/media/1043/2019-jamming-spoofing-of-gnss.pdf).

On the system side, one possibility would be to apply advanced frequency hopping spread spectrum techniques where the signal is rapidly switching transmitting signals as well as appropriate anti-jamming methods (see e.g. Gao et al. 2018). Of course, increasing the transmitting satellite signal power by a large number of dBs (see e.g. GLONASS-K2 power capacity 4370 W) would be the best possible anti-jamming method, however, it requires larger satellites and violates the ITU (International Telecommunication Union) conventions and rules when using the high power.

From the above we may conclude that we have to meet challenges in future in improving not only the receivers but also the satellites with respect to anti-jamming, anti-spoofing and other cyber-attacks (Harrison et al. 2020; Wang et al. 2020).

*Interoperability*^2^ in its most strict sense assuming same center frequencies for signals (H/W) but allowing different codes (S/W) and a different reference system both in time and coordinates has the great user advantage to have a simple receiver for tracking signals from several satellite navigation systems. However, also here we may come to a limit. We are increasing the internal satellite noise floor to a level where we could get problems in acquiring signals with a normal receiver (Hein 2010). Interoperability has another advantage for the user: it forces the systems to take up improvements coming from the other ones. The market will only consider a system in a navigation chip if it is comparable in quality to the other ones. Otherwise it would disregard systems which cannot possibly contribute in a combination approach. The progressive digitalization of both, the satellite navigation payload and the user receiver including the front-end may change the strict hardware requirements for interoperability in near future, in particular assuming identical center frequencies.

## New opportunities and perspectives

### Advanced Receiver Autonomous Integrity Monitoring (ARAIM) and SBAS

It is generally recognized that ARAIM has a great potential for SBAS (EU-US Cooperation on Satellite navigation 2015; EU-US Cooperation on Satellite navigation 2016; Fernández et al. 2019). Horizontal ARAIM is expected to be available around 2023 and vertical ARAIM following a few years later. SBAS systems are guaranteed until 2035, especially for aviation (http://www.faa.gov). But what happens after 2035? Will SBAS systems become obsolete?

### Potential of 5G wireless networks

**Fig. 7:**
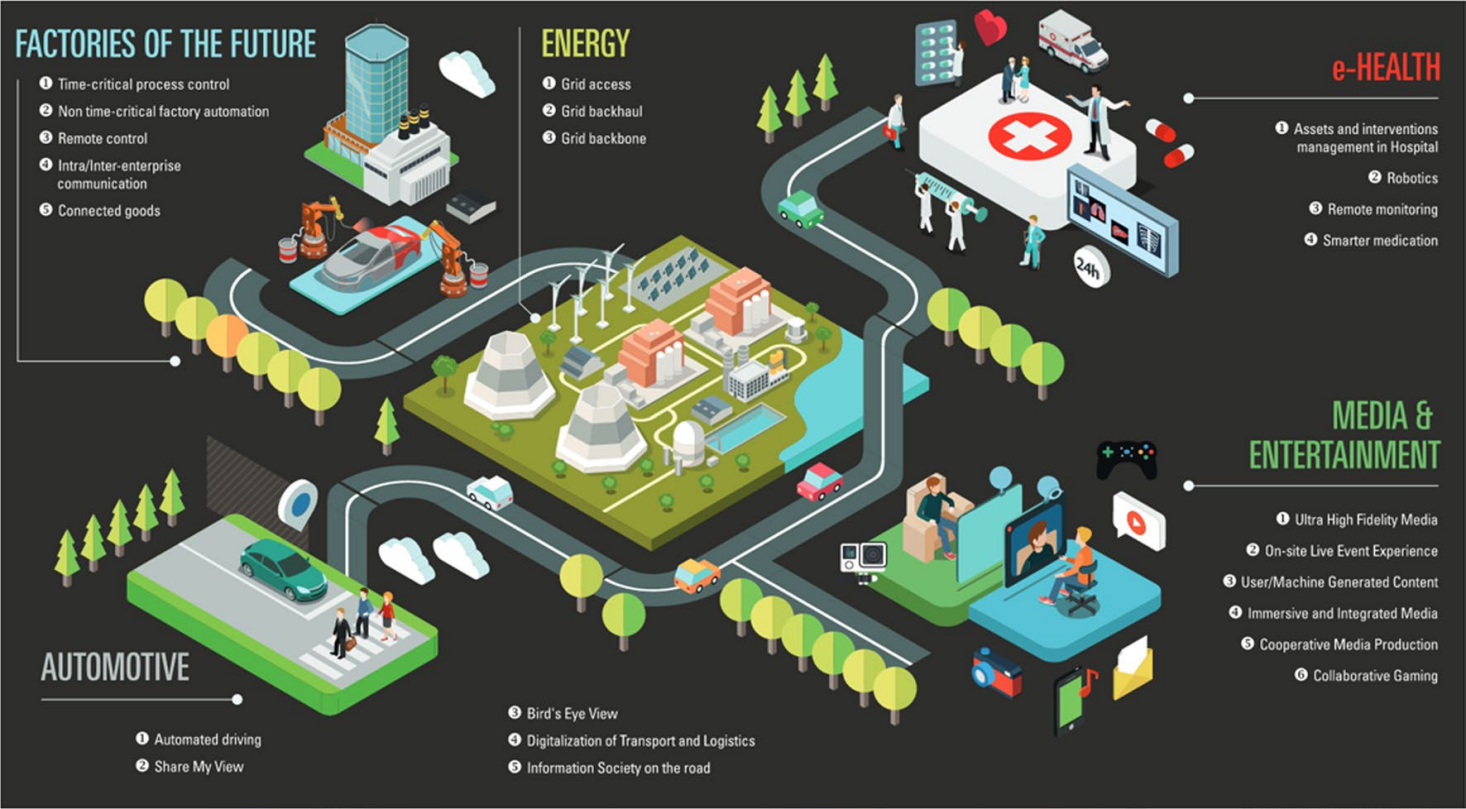
5G wireless networks applications—Reference Prieto-Cerdeira et al. (2018)

Introduction of 5G wireless networks is expected after 2020 (Fig. 7). The standardisation process for the first release incorporating 5G capabilities was completed in June 2018 with 3GPP Release 15. Phase 2 is about to be completed. 5G technology with its many new mission-critical services and positioning applications may represent a new mobile revolution in the wireless landscape. The main targets include the Internet of Things (IoT) and ultrafast enhanced mobile broadband using millimeter wave bands and small cells. The standardized positioning levels of 3GPP can be found in Prieto-Cerdeira et al. (2019, Table 2). A competitor of our GNSS? Or will the number of GNSS applications decrease? Or, most likely, a hybridization/fusion GNSS/5G will start to develop for certain applications.

**Fig. 8:**
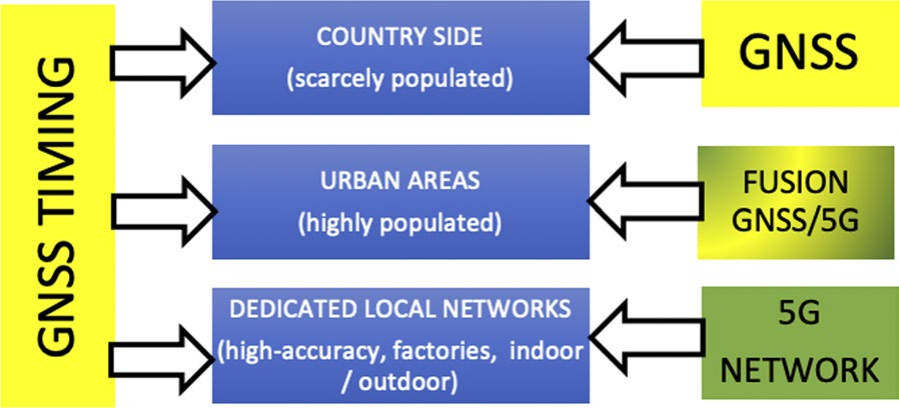
Applications of 5G wireless networks and GNSS

In the following there will be a short discussion what the role of GNSS and the one of 5G most likely will be in future (Cozzens 2019; Kishiyama et al. 2017; Prieto-Cerdeira et al. 2018, 2019) (Fig. 8).

*5G Timing by GNSS* The high-performance mobile services delivered over the 5G networks are extremely dependent on precise time from GNSS so they can synchronize radios, enable new applications and minimize interference.

*GNSS in areas with scarce population* The high accuracy of 5G networks can be only realized using many dense base stations. Due the commercial character of the operating companies, this will be only the case where the population is high—certainly not in the country side.

*Dedicated 5G networks for large companies and production* In order to become independent by the telecom operators and aiming for the highest 5G positioning cm/mm accuracy in- and outdoor for their production, large factories intend to install and operate their own very local 5G network with dense base stations. Here GNSS may be replaced by 5G (except GNSS timing).

*Fusion of GNSS and 5G in urban areas* Due to the fact that GNSS may have a downgraded accuracy in urban canyons caused by limited satellite availability, unfavorable satellite geometry and multiple multipath, a fusion of the 5G cm-wave with GNSS might result in higher positioning accuracies (Peral-Rosado et al. 2018). Therefore, compatibility and interoperability of 5G and GNSS is necessary.

### Satellite navigation and new space (Hein 2018; Reid et al. 2018)

In the last years, a move in space technology came up, called “New Space”. Although there is no unique definition, it is certainly a movement and new philosophy, encompassing a globally emerging, private spaceflight and aerospace industry which is more socio-economically-oriented. In other words, working commercially and independent of governmental-funded (political) space programs with a faster, cheaper and better access to space.

In a wider definition of New Space, new business models and new manufacturing processes building up on alternative methods are considered in addition (ESA Space 4.0).

Examples of New Space systems might be the Low Earth Orbit (LEO) systems with many hundreds or even thousands of mini-satellites mainly dedicated for communication and internet. OneWeb (https://onewebsatellites.com) which has been aiming to launch at least 648 satellites to deliver global broadband connectivity, has 74 satellites in orbit.^3^ SpaceX Starlink (https://www.spacex.com/webcast) is currently being built-up. SpaceX’s deployed 60 Starlink satellites in orbit after a successful launch on April 22, 2020 bringing the broadband internet project to more than 420 satellites. The first phase of the Starlink network will include 1584 satellites orbiting about 550 km above Earth in planes inclined 53 degrees to the equator. That part of the constellation SpaceX intends to launch through the end of 2020. (https://www.nzz.ch/wissenschaft/starlink-so-funktioniert-das-satelliteninternet-von-elon-musk-ld.1493375).

Amazon’s project Kuiper (https://www.geekwire.com/2019/amazon-project-kuiper-broadband-satellite) will move in 2020 to a permanent research and development headquarter with state-of-the-art facilities for the design and testing of its planned mega-constellation of 3236 LEO satellites in altitudes of 590/609/629 km for low-latency, high-speed broadband. Telesat Canada (https://www.telesat.com/news-events) has similar plans for broadband communications scheduled to start operations from their LEO satellites (first Phase 1 LEO satellites were launched in 2018).

#### But, can those LEO systems be used for satellite positioning and navigation?

Some quick considerations: GPS signals broadcast at 27 Watts which are received at 158 × 10^−18^ Watts on Earth. LEO signals of Starlink are 1000 × (30 dB) stronger compared to MEO (GNSS). But it takes 7 LEOs to match the coverage of 1 MEO.

200 + LEOs are needed for similar coverage—no problem, all mentioned LEO systems have significantly more than 200 satellites. Consequently, the geometry (Dilution of Precision—DOP values) is three times better than that of present GNSS. Considering further that a positioning error is approximately Signal-in-Space (SIS) User Range Error (URE) x geometry, it becomes clear that the LEO system’s geometry is three times better and relaxes the URE. A constellation like SpaceX Starlink could have three times worse URE and still reaches a positioning performance comparable to GPS (about 3 m horizontally, 4–5 m vertically).

The chip-scale atomic clocks (low power < 120mW, small size 17 cc volume, low-cost < 1000 USD … 300 USD) in the LEO satellites are approximately 100 × worse at one day compared to GPS atomic clocks. However, we may get comparable performance if they were updated once per LEO orbit (approx. every 100 min) instead of once per 12 h (GPS). Simple computations of LEO orbits by ground stations indicate that it is possible to achieve 3 m RMS, if using in addition cross-links even approximately 1.5 m.

#### What about costs? No taxpayer’s money has to be provided by governments…?

One can only speculate whether or not all LEO systems for satellite communications and internet mentioned above will be actually realised. As a result, tremendous competition for market shares would ensue between the companies, also affecting terrestrial communication, in particular 5G Wireless Networks. Also, I would not expect the various companies to modify their payloads to include satellite navigation as discussed above.

**Fig. 9:**
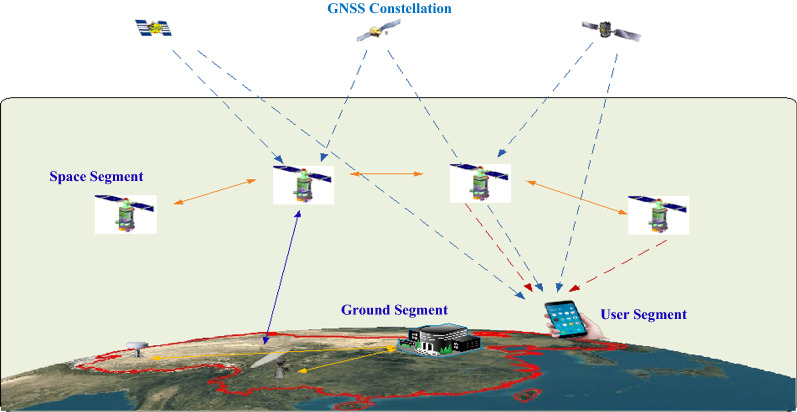
The Centispace-1 LEO satellite-based augmentation system. Reference: Yang (2019)

However, the Beijing Future Technology Company (Su et al. 2019; Yang 2019) is planning, developing and will operate a LEO satellite-based augmentation system to the MEO GNSS, called Centispace-1 (Fig. 9). Small satellites with a weight of approx. 100 kg in a Walker constellation 120/12/0, an altitude of 975 km and an inclination of 55° should receive GNSS from the MEO satellites and transmit in GNSS L1/L5 interoperable frequencies. High-speed crosslinks between the satellites are designed. The launch of a first experimental satellite happened already 2018, five experimental satellites will follow in 2020. Between 2021 and 2023 120 operational satellites will be launched and the ground segment finalised. Centispace-1 will deliver high accuracy and service of the order of 50 cm and an integrity service with an alarm time < 3 s and 99.99% global availability. In the combined processing with MEO GNSS data a point positioning < 10 cm with a significantly smaller convergence time of less than 1 min (due to the high doppler of the LEO satellites) is expected.

However, these will be not the latest developments over the next years. The Cubesat technology and many low-cost low power miniaturized sensors fitting on them will enable many new IoT applications as well as the LEO augmentation of various MEO GNSS.

### Megatrends in satellite navigation

*Global navigation satellite systems* As mentioned above, all of the four GNSS will be fully operational available by the end of 2020/beginning of 2021. The Chinese BDS, also the last one which started with the developments, is the most advanced: It is currently the only one which has a regional part with IGSO satellites (which will be also used for the transmission of SBAS messages) and it will be already extended by a LEO component called Centispace in the next years which significantly improves the convergence time of high-precision absolute positioning.

GPS III will improve its robustness over the next years whereas Galileo still has to prove it (in particular after the long outage in 2019). ESA has studied a regional aspect of IGSO satellites over Europe with regard to the evolution of the system. However, it is not yet decided whether it will be realised with the second generation of Galileo after 2025. The Russian GLONASS system has similar plans (GLONASS-B). What is even more needed, however, is a globally distributed ground GLONASS control system.

*Regional navigation satellite systems* The South Korean KPS will be developed over the next decade—overlapping the Japanese QZSS system which will be further expanded to 7 satellites.

*Satellite*-*Based Augmentation Systems (SBAS)* It is expected that after the first dual-frequency dual-system EGNOS V3, also Russia and China will incorporate in their SBAS their own GNSS (GLONASS and BDS, respectively) in addition to GPS. Whereas the SBAS in South Korea, in Russia, Australia and China are still being developed, and a guarantee of the availability of SBAS for civil aviation is guaranteed till 2035, is ARAIM showing already its large potential for providing Cat-I integrity similar to SBAS. Horizontal ARAIM will be available in the next 3–4 years and vertical ARAIM might come by the end of this decade. Will it replace then the SBAS after 2035?

*CubeSats, mini*- *and nano*-*satellites* The potential of CubeSats and the availability of miniaturized, low-power and low-cost sensors for those mini- or nano-satellites in LEO is increasing with every day, see e.g. https://www.nanosats.eu, https://www.esa.int/Enabling_Support/Space_Engineering_Technology/Technology_CubeSats, https://www.nasa.gov/mission_pages/cubesats/index.html. Thus, many IoT and other Earth observation applications become possible on a regional scale with a relatively small budget. CubeSats have passed the time where they were only considered as an educational tool for universities. The expensive space hardening of the payload is replaced by cheaper smart (redundancy) techniques. CubeSats will form space augmentations in LEO to the present GNSS over the next years. However, also exploration to Moon, Mars and other planets will take advantage of it. Corresponding studies are already running. We will see soon GNSS beyond the Earth up to the moon and further in space (https://www.esa.int/Enabling_Support/Space_Engineering_Technology/Winning_plans_for_CubeSats_to_the_Moon).

*Digitalisation* will be considered in GNSS payloads enabling on-orbit reprogramming of GPS signals and transmissions and *artificial intelligence* in space traffic management.

*Quantum communications* will contribute to a more reliable and trustworthy satellite navigation. Quantum communication takes advantage of the laws of quantum physics to protect data. These laws allow particles—typically photons of light for transmitting data—to take on a state of superposition, which means they can represent multiple combinations of 1 and 0 simultaneously. The beauty from a cybersecurity perspective is that the transmission of highly sensitive data by quantum communication is ultra-secure.

In the next years we will see many projects addressing one of the main challenges of satellite navigation: *GNSS safety and security (space cyber security)*. In the past years, our society and economy have become largely dependent on GNSS, computer networks and Internet of Things (IoT) solutions. This has led to a significant growth of cyber-attacks. Big data, virtual and augmented reality and artificial intelligence will even create more cyber risks. This evolving environment presents new opportunities for the space industry to come up with new commercial cyber security solutions.

*GNSS receiver* Although the H/W and S/W tools, like the inertial navigation system on a chip, the chip-scale atomic clock, the phased array antenna, detection/mitigation techniques for interferences are developed and jamming and spoofing may be happening, is the consideration of those tools in the civilian receivers still rare. Smartphones have seen some progress, which are nowadays equipped with almost all GNSS and RNSS. Android phones provide the capability to use GNSS raw data and can use self-developed software for specific user applications. It is only to be expected that more and more sensors combining various navigation methods will be implemented over time.

*5G wireless networks* Assuming a dense network of base stations, wireless 5G is able to provide centimeter navigation—however, only on a local scale. Will it be substituting or complementing/locally augmenting the global GNSS—as predicted in Fig. 8? Interesting developments—to be carefully followed and monitored.

*Fighting with Space Debris* As mentioned above, thousands of satellites will be launched in the coming years. The International Space Station (ISS) had to change its course often in the past in order to avoid getting seriously damaged by space debris and other satellites. Therefore, space traffic management studies have started at ESA and will intensively continue over the next decade Navigation of satellites will play an important role (https://www.esa.int/Safety_Security/Space_Debris).

### Some remarks

1. Although we had to think in longer timeframes considering the developments of GNSS (which took at the beginning almost two decades for a system) it is hard to predict the future of satellite navigation. Like computers GNSS receivers are depreciated over a time of three years. It is therefore understandable that a forecast for more than a few years is almost impossible.
2. If we look to the future of GNSS and RNSS, we have to accept:
  The signal is weak… The signal is easily jammed…The signal can be spoofed… The signal is subject to atmospheric perturbations…The signal doesn’t penetrate buildings…The signal has problems with urban and natural obstructions…
  But is there a real substitute or alternative to GNSS?
  Back-up by eLoran? Iridium NEXT?
  Chip-scale atomic clocks, other terrestrial systems?
  Map matching, radar, lidar, vision?
  Com cell-id, 5G, INS, WiFi?
  However, none of the above are also all-weather systems, have excellent accuracy, global coverage, high reliability, low cost, low complexity, minimal infrastructure needs, versatility.
3. Satellite navigation systems are not like other space projects serving only small scientific communities and last only for a few years. They are serving every citizen with Positioning, Navigation and Time (PNT). PNT is never the primary product; it is an enabler for many value-added applications. The critical infrastructure of many states depends already on GNSS. After more than two decades of building up the satellite systems, satellite navigation will stay many decades….
4. To what extent there will be an impact of the worldwide coronavirus pandemic and the subsequent crisis in economy is currently (April 2020) unclear. So far, we have seen delays in satellite launches, space projects and OneWeb’s filing for bankruptcy.

## Footnotes

1 Compatibility refers to the ability of space-based positioning, navigation, and timing services to be used separately or together without interfering with each individual service or signal, and without adversely affecting national security. (NSPD-39: U.S. Space-Based Position, Navigation, and Timing Policy, December 15, 2004).

2 *Interoperability* refers to the ability of civil space-based positioning, navigation, and timing services to be used together to provide better capabilities at the user level than would be achieved by relying solely on one service or signal. (NSPD-39: U.S. Space-Based Position, Navigation, and Timing Policy, December 15, 2004).

3 OneWeb filed for Chapter 11 bankruptcy on 28 March 2020. Other LEO systems like Iridium, Globalstar, Orbcomm and Teledesic all went bankrupt about two decades ago, though only Teledesic failed to emerge from bankruptcy and deploy a second-generation constellation (Henry 2020).

## Availability of data and material

Data sharing is not applicable to this article as no datasets were generated or analysed in this review article.
